# Subcellular origin of mitochondrial DNA deletions in human skeletal muscle

**DOI:** 10.1002/ana.25288

**Published:** 2018-08-21

**Authors:** Amy E. Vincent, Hannah S. Rosa, Kamil Pabis, Conor Lawless, Chun Chen, Anne Grünewald, Karolina A. Rygiel, Mariana C. Rocha, Amy K. Reeve, Gavin Falkous, Valentina Perissi, Kathryn White, Tracey Davey, Basil J. Petrof, Avan A. Sayer, Cyrus Cooper, David Deehan, Robert W. Taylor, Doug M. Turnbull, Martin Picard

**Affiliations:** ^1^ Wellcome Centre for Mitochondrial Research and Newcastle Centre for Ageing and Vitality Institute of Neuroscience, Newcastle University Newcastle upon Tyne United Kingdom; ^2^ Institute of Neurogenetics University of Lübeck Lübeck Germany; ^3^ Molecular and Functional Neurobiology Group Luxembourg Center for Systems Biomedicine, University of Luxembourg Esch‐sur‐Alzette Luxembourg; ^4^ Department of Biochemistry Boston University School of Medicine Boston MA; ^5^ Electron Microscopy Research Services Newcastle University Newcastle upon Tyne United Kingdom; ^6^ Meakins‐Christie Laboratories, Department of Medicine McGill University Health Centre Montreal Quebec Canada; ^7^ National Institute for Health Research Newcastle Biomedical Research Centre Newcastle upon Tyne Hospitals National Health Service Foundation Trust and Newcastle University Newcastle upon Tyne United Kingdom; ^8^ Medical Research Council Lifecourse Epidemiology Unit University of Southampton Southampton United Kingdom; ^9^ Institute of Cellular Medicine Newcastle University Newcastle Upon Tyne United Kingdom; ^10^ Department of Psychiatry, Division of Behavioral Medicine Columbia University Medical Center New York NY; ^11^ Department of Neurology and Columbia Translational Neuroscience Initiative H. Houston Merritt Center, Columbia University Medical Center New York NY; ^12^ Columbia University Aging Center Columbia University New York NY

## Abstract

**Objective:**

In patients with mitochondrial DNA (mtDNA) maintenance disorders and with aging, mtDNA deletions sporadically form and clonally expand within individual muscle fibers, causing respiratory chain deficiency. This study aimed to identify the sub‐cellular origin and potential mechanisms underlying this process.

**Methods:**

Serial skeletal muscle cryosections from patients with multiple mtDNA deletions were subjected to subcellular immunofluorescent, histochemical, and genetic analysis.

**Results:**

We report respiratory chain–deficient perinuclear foci containing mtDNA deletions, which show local elevations of both mitochondrial mass and mtDNA copy number. These subcellular foci of respiratory chain deficiency are associated with a local increase in mitochondrial biogenesis and unfolded protein response signaling pathways. We also find that the commonly reported segmental pattern of mitochondrial deficiency is consistent with the three‐dimensional organization of the human skeletal muscle mitochondrial network.

**Interpretation:**

We propose that mtDNA deletions first exceed the biochemical threshold causing biochemical deficiency in focal regions adjacent to the myonuclei, and induce mitochondrial biogenesis before spreading across the muscle fiber. These subcellular resolution data provide new insights into the possible origin of mitochondrial respiratory chain deficiency in mitochondrial myopathy. Ann Neurol 2018;84:289–301

Nuclear‐encoded mutations affecting the replication and maintenance of the mitochondrial genome (mtDNA) are a common cause of mitochondrial disease,^1^ typically causing multiple mtDNA deletions. Due to the polyploid nature of mtDNA in individual cells, mtDNA deletions must clonally expand to high levels before they cause respiratory deficiency. The clinical phenotype for these disorders is variable, ranging from isolated progressive external ophthalmoplegia (PEO) to PEO with additional symptoms including proximal myopathy, ataxia, sensory axonal neuropathy, and optic atrophy. Genes associated with these phenotypes encode proteins involved in mtDNA replication (eg, *POLG, TWNK, DNA2, MGME1*), deoxyribonucleoside triphosphate supply (eg, *TP, RRM2B, TK2, DGUOK*), and proteins regulating mitochondrial dynamics (*OPA1, MFN2*).^2^

Clonal expansion of mtDNA deletions is an important cause of mitochondrial respiratory chain deficiency. A critical unanswered question is where and how, within the cell cytoplasm, new mtDNA deletions expand from a single mutated mtDNA molecule to become the dominant mtDNA species. Several theoretical models have been proposed to explain the clonal expansion of mutant mtDNA.^3^, ^4^, ^5^, ^6^, ^7^, ^8^ However, previous models were based on cell populations in vitro^9^, ^10^ or invertebrate models with pre‐existing mixtures of mutant mtDNA.^11^, ^12^

Skeletal muscle fibers have a highly organized cytoarchitecture, where spatially restricted mitochondrial subpopulations exist.^13^, ^14^ Subsarcolemmal (SS) mitochondria are located at the periphery of the muscle fiber adjacent to the sarcolemma membrane, a subset of which are perinuclear; whereas intermyofibrillar (IMF) mitochondria are located between the myofibrils at the Z‐band.

This raises the question of how clonal expansion proceeds through these highly organized and spatially restricted mitochondrial populations. To investigate this process, we sought to localize and analyze the earliest visible biochemical defect in muscle fibers from patients. Increasing our understanding of this phenomenon will provide insight into the origins of mtDNA deletions and respiratory chain deficiency, with potential diagnostic and prognostic implications.

## Materials and Methods

### Muscle Biopsies

Muscle biopsies from quadriceps were obtained via needle biopsy under local anesthesia. Ethical approval was granted by the Newcastle and North Tyneside local research ethics committees (reference 2002/205), and prior informed consent was obtained from each participant. Normal human ageing samples were obtained from the Hertfordshire cohort.^15^ Control tissue (n = 20) was acquired with prior informed consent from people undergoing anterior cruciate ligament surgery, following approval by the Newcastle and North Tyneside local research ethics committees (reference 12/NE/0395). All experiments were carried out in accordance with the approved guidelines. Muscle samples were frozen in liquid N_2_‐cooled isopentane, mounted in OCT, and stored at −80 °C until use, or fixed for electron microscopy.

All biopsies from mtDNA maintenance disorders (n = 7) had confirmed genetic diagnoses. Sequential cytochrome c oxidase (COX)/succinate dehydrogenase (SDH) histochemistry was used to screen biopsy specimens, and those with a suitable muscle histology and a significant degree (>2–5%) of COX deficiency (COX‐SDH+) were included (Supplementary Table). Patient selection for each experiment was based on tissue availability due to the large number of sections required for each experiment. Genetic and immunofluorescent analyses were completed on separate sections to enable high resolution quantitative fluorescent imaging in parallel with high contrast accurate laser captue microdissection.

### COX/SDH Histochemistry

To quantify the prevalence of COX‐deficient foci, cryosectioned muscle (10 μm) from transversely orientated muscle blocks, from mtDNA maintenance disorder patients (n = 6, Patients 1–4, 6, and 7), was subjected to sequential COX/SDH histochemistry^16^ and independently analyzed by 2 investigators. Strict criteria were applied for foci quantification. Only circumscribed foci deficient in COX activity but with SDH activity (ie, COX‐SDH+) that were localized in an otherwise COX‐positive fiber were counted as a COX‐deficient niche.

### COX/SDH and Nuclear Staining

Serial 8 µm sections were subject to COX/SDH histochemistry before counterstaining with 4,6‐diamidino‐2‐phenylindole (DAPI; D9542; Sigma, St Louis, MO; 2 μg/ml) for 5 minutes and mounting in ProLong Gold (P10144; Thermo Fisher Scientific, Waltham, MA). Images were captured in both brightfield and DAPI channels individually and merged. At least 14 serial sections were reconstructed to provide 3‐dimensional information about the length of COX‐deficient foci and their proximity to myonuclei.

### mtDNA Deletion and Copy Number Analysis

Serial muscle sections (15 μm) treated for COX/SDH histochemistry were cryosectioned onto PEN membrane slides (#000635‐17; Zeiss, Oberkochen, Germany). Patient selection for genetic analysis was based on tissue availability. Muscle fibers with regions of focal cross‐sectional COX deficiency were identified, and the COX‐deficient area was laser microdissected and captured into separate 0.2ml tubes using a PALM system (Zeiss). Two identically sized subsarcolemmal regions from the remainder of the muscle fiber were also captured, in addition to whole COX‐positive and COX‐deficient muscle fibers as controls. Muscle fibers were lysed in 15 µl single‐cell lysis buffer (0.5M Tris‐HCl, 0.5% Tween 20, 1% proteinase K, pH 8.5) and incubated at 55 °C for 3 hours followed by 10 minutes at 95 °C. The lysate was then diluted 1:5 and analyzed in triplicate using a D‐Loop/*MT‐ND1*/*MT‐ND4* triplex real‐time polymerase chain reaction (PCR) deletion assay.^17^ A serial dilution of the p7D1 plasmid was used to generate a standard curve from each plate. For a plate of samples to be included in analyses, an amplification efficiency of 100 to 95% was required for the standard curve. Major arc deletions were quantified by computing the ratio of *MT‐ND1*/*MT‐ND4*. Negative values arising from this assay, which may result from deletion of *MT‐ND1*, are shown as zero.

Due to the low mtDNA content of the foci and of the similarly sized COX‐positive regions of the muscle fiber, we further implemented a stringent quality control threshold requiring all foci and corresponding regions of the same fiber to be at least three quantification cycles values below the no‐template control for *MT‐ND1*. The D‐Loop region can become triple‐stranded during transcription/replication,^18^ so analysis of D‐Loop/*MT‐ND1* ratios in cells that did not have deletions encompassing *MT‐ND1* were used to exclude this possibility. As a control, 2 to 4 fully COX‐deficient and COX‐positive control fibers were also run in triplicate on every plate.

By these criteria, ∼68% of cells were eliminated due to inadequate amplification. Overall, 27 foci and their matched COX‐positive regions from 5 patients with *POLG* (n = 2), *RRM2B* (n = 2), and *TWNK* (n = 1) mutations (Patients 2–5 and 7; see Supplementary Table) showed robust amplification and were used for subsequent analyses.

### Immunofluorescence

Serial cryosections (8 μm) were subject to quantitative immunofluorescence staining.^19^ Antibodies used include anti–mitochondrial cytochrome oxidase I (MTCOI; ab14705; Abcam, Cambridge, MA), anti–Succinate Dehydrogenase complex subunit A (SDHA; Abcam ab14715), anti‐laminin (Sigma L9393), anti‐mouse IgG2a‐488 (S‐21131), anti‐rabbit 405 (A‐31556), biotinylated anti‐mouse IgG1 (S32357), and streptavidin‐647 (S21374; all from Life Technologies, Carlsbad, CA). Following the secondary antibody incubation, sections were washed and counterstained with DAPI (2 μg/ml) for 5 minutes and mounted in ProLong Gold. Sections were imaged on a Zeiss Axio imager M1 microscope equipped with a motorized stage using multidimensional acquisition tiling in ZEN (Zeiss, blue edition).

SDHA expression was correlated with porin (VDAC1), a known indicator of mitochondrial mass,^19^ with significant concordant SDHA/porin levels indicating that SDHA is a viable mitochondrial mass marker.

### Image Analysis

The average fluorescent intensity corresponding to the abundance of an investigated protein in the muscle fibers was quantified using Image J (version 1.50i) or IMARIS v.7.7.2 (Bitplane).^19^, ^20^ The “plot profile” functions in Image J and ZEN (blue edition) were used to compare changes in MTCOI and SDHA abundance across muscle fibers, subtracting the background fluorescence for each channel.

### Immunofluorescent Assessment of Signaling Proteins

Antibodies against PGC1α (Ab3243; Millipore, Billerica, MA), transcription factor A, mitochondrial (TFAM; Abcam Ab119684), NEF2L2 (Abcam ab31163), ClpP (Sigma HPA010649), Htra2 (AF1458; R&D Systems, Minneapolis, MN), mtHsp70 (Thermo Fisher Scientific), Hsp60 (611562; BD Biosciences, Franklin Lakes, NJ), GPS2,^21^ p62 (Progen GP62‐C), Beclin1 (Millipore Ab15417), Pink1 (Abcam Ab23707), LC3‐II (4108; Cell Signaling Technology, Danvers, MA), and parkin (sc‐32282; Santa Cruz Biotechnology, Santa Cruz, CA) to assess retrograte signaling factors and mitophagy markers were used in combination with MTCOI, SDHA, and DAPI to assess changes in relation with COX deficiency in single muscle sections. Antibodies that did not produce a sufficient signal‐to‐noise ratio, or were highly correlated with mitochondrial mass, were removed from further analysis. TFAM, Hsp60, GPS2, and Beclin1 were selected for immunofluorescence on serial sections (n = 4) of patients with multiple mtDNA deletions (n = 3, Patients 4, 5, and 7; see Supplementary Table). IMARIS v.7.7.2 (Bitplane) was used to assess average fluorescent intensity of signaling markers relative to MTCOI/SDHA ratio in whole COX‐positive and COX‐deficient fibers. Average intensity of the foci was compared to a COX‐positive region of the fiber.

### Fiber Perimeter Profiling to Identify Perinuclear Domains and Regions of Mitochondrial Deficiency

Serial skeletal muscle sections were labeled for MTCOI, SDHA, and DAPI (as described above) from 3 patients with multiple mtDNA deletions (Patients 3, 5, and 7; see Supplementary Table). A total of 74 fibers containing foci of mitochondrial deficiency were identified by visual inspection and used for fiber section perimeter profiling. DAPI, SDHA, and MTCOI fluorescence intensity profiles were constructed. Differences in DAPI intensities between sections were minimized by subtracting the background intensity (modal, unsaturated DAPI intensity) of each section. Perinuclear perimeter domains were identified as areas with a corrected DAPI intensity greater than the 0.85 quantile of corrected intensity observed in all 74 fibers across serial sections in 3 patients. All points along intensity profiles were manually annotated as COX‐positive or COX‐deficient by visual inspection of perimeter profiles as well as linear line scans through the center of the muscle fiber to identify perimeter segments with mitochondrial deficiency.

### Predicted and Observed Levels of Focus–Perinuclear Overlap

For each section, we measured the fraction of the perimeter annotated as perinuclear (P), the fraction of the perimeter annotated as focal deficiency (F), and the fraction of the perimeter annotated as focus‐perinuclear overlap (O). We also calculated the fraction of the perimeter that we would expect to be annotated as focus–perinucleus overlap if the locations of the perinuclear region and nuclei were unrelated and random (overlap predicted [O_pred_]). According to the specific multiplication rule for the probability of 2 independent events co‐occurring, we would expect that:

$$O_{\text{pred}}=P\times F$$

To test whether differences between O_pred_ and observed O_obs_ were significant, a 1‐tailed *t* test to examine whether O_pred_ − O_obs_ was significantly different from zero was performed. Code and data underlying profiling and overlap analysis can be found at https://github.com/lwlss/MitoDysfunctionFoci.

### Serial Block Face Scanning Electron Microscopy

As described previously, serial block face scanning electron microscopy (SBF‐SEM) sample preparation and imaging were performed^22^ to examine the three‐dimensional (3D) organization of intermyofibrillar mitochondria. Four muscle fibers from 12 muscle biopsies were imaged and 3D models assessed to determine mitochondrial distribution and anisotropy.

### Statistics

A combination of 2‐tailed unpaired (whole muscle fibers) and paired (matched subcellular regions) *t* tests was used. Statistical significance was set at 0.05. Data for focal regions of deficiency are presented as individual COX‐deficient fiber regions with paired COX‐positive regions. All analyses were performed in Prism v7.0 (GraphPad Software, San Diego, CA).

## Results

### Foci of Respiratory Chain Deficiency Are a Pathological Hallmark of mtDNA Deletions

**Figure 1:**
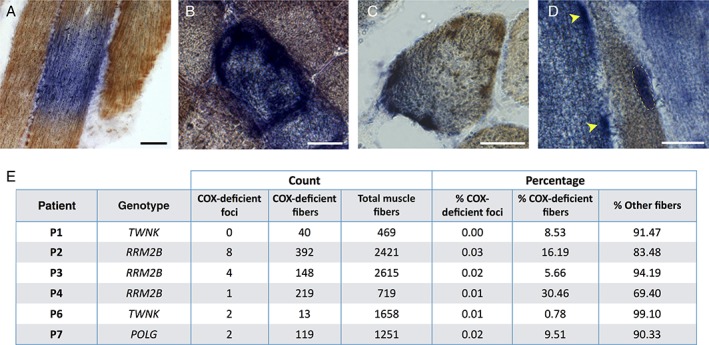
Subcellular localization of respiratory chain dysfunction in human skeletal muscle. (A) Three skeletal muscle fibers in longitudinal orientation from a cryosection subjected to cytochrome c oxidase (COX, brown) and succinate dehydrogenase (SDH, blue) histochemistry to detect COX and SDH activity. The central fiber has segmental COX deficiency, indicative of segmental distribution of mtDNA defects. (B) Skeletal muscle fiber in cross‐sectional (ie, transverse) orientation that is fully deficient for COX activity and positive for SDH activity. (C) A focal region of COX deficiency in cross‐sectional orientation. (D) In longitudinal orientation, focal region of COX deficiency in a case of multiple mtDNA deletions. Note the restricted nature of COX deficiency consistent with a perinuclear region *(dotted line)* and the subsarcolemmal increase in SDH activity in an adjacent fully COX‐deficient fiber *(arrowheads)*. (E) Summary of quantitative analysis of foci frequency in mtDNA maintenance disorders (n = 6). Count data for total, COX‐deficient SDH‐positive fibers (COX‐deficient fiber) and COX‐deficient foci are presented for each case followed by a percentage of fibers classified as COX‐deficient fiber, COX‐deficient foci, or other (COX‐positive or intermediate COX deficiency). Scale bars = 25 μm.

We investigated skeletal muscle biopsies from patients with multiple mtDNA deletions due to nuclear gene mutations affecting mtDNA maintenance (n = 7; see Supplementary Table, Fig 1). The commonly reported pattern of respiratory chain deficiency in skeletal muscle consists of a mosaic pattern of COX‐positive and COX‐deficient muscle fibers, with the affected muscle fibers observed in the longitudinal orientation harboring confined segments of respiratory chain deficiency (see Fig 1A).^3^, ^23^, ^24^, ^25^, ^26^

As expected, we observed COX‐deficient muscle fibers in both the transverse and longitudinal orientations (see Fig 1A, B). In addition, we identified focal regions of respiratory chain deficiency within individual muscle fibers (see Fig 1C, D). These foci, which had not been previously reported, were observed in patients with mitochondrial disease and normal muscle biopsies of older individuals, but not in age‐matched control biopsies (n = 20). COX‐deficient foci were also observed in muscle biopsies of older individuals (i.e., normal aging).

To estimate the prevalence of COX‐deficient foci in specific mtDNA maintenance disorders, we surveyed cryosections reacted for sequential COX/SDH histochemistry from patients with *TWNK, RRM2B*, and *POLG* mutations (n = 6, Patients 1–4, 6, and 7). A total of 11 COX‐deficient foci and 929 fully COX‐deficient muscle fibers were identified (see Fig 1E). Skeletal muscle biopsies contained on average 11.9% (range = 0.8–30.5%) fully COX‐deficient muscle fibers, compared to 0.15% (range = 0–0.33%) with restricted COX‐deficient foci (see Fig 1E). The total prevalence of COX‐deficient foci relative to total muscle fibers and fully COX‐deficient muscle fibers was estimated at 1:571 and 1:58, respectively.

### Focal Regions of Deficiency Are Restricted to the Subsarcolemmal Space

**Figure 2:**
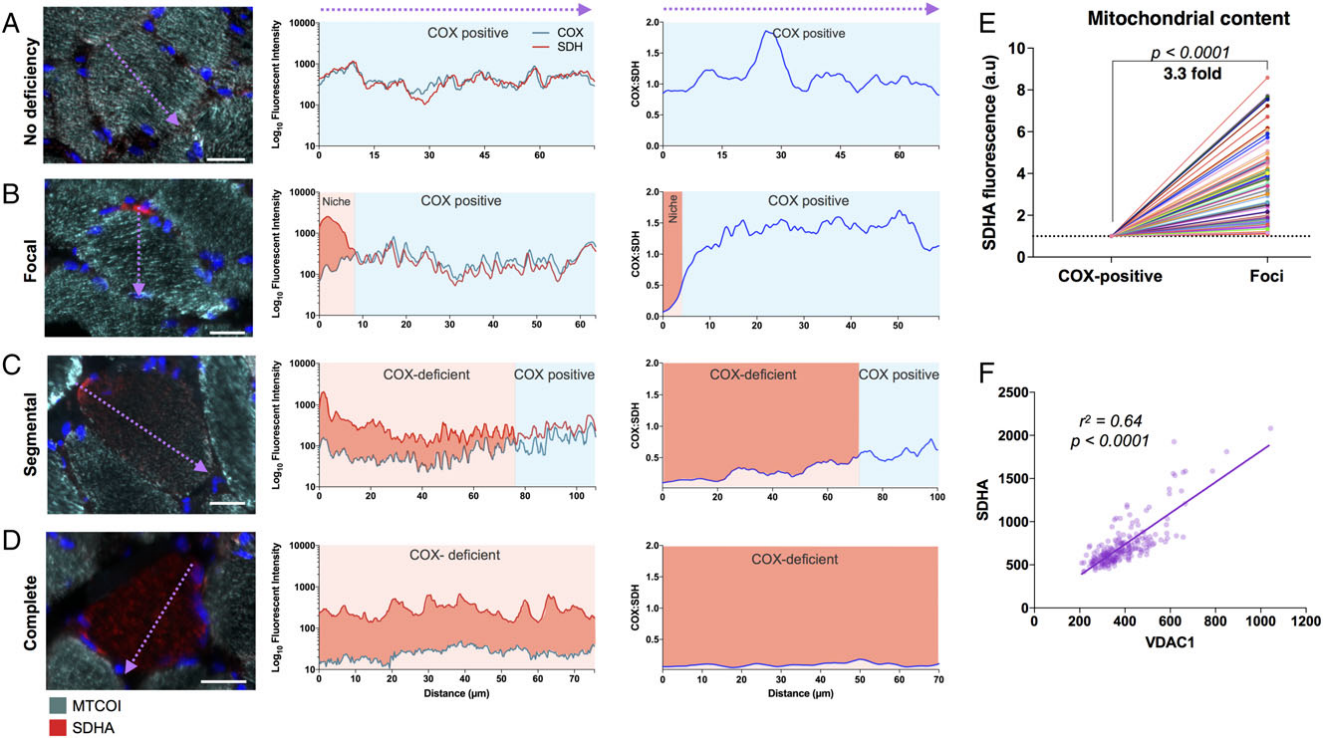
Foci distribution of cytochrome c oxidase (COX) deficiency and mitochondrial content by immunofluorescence in human muscle fibers. (A) Mitochondrial cytochrome c oxidase subunit I (MTCOI; green), succinate dehydrogenase (SDH) complex subunit A (SDHA) (red), and nuclei (blue) immunofluorescent labeling and quantification of a normal COX‐positive skeletal muscle fiber (Patient 7, *POLG*; left). Corresponding fluorescence intensity profile along the diameter denoted by the arrow (middle), and COX:SDH ratio (right). (B) Muscle fiber (Patient 7, *POLG*) with a niche of focal COX deficiency denoted by the red area denoted in the fluorescence intensity plots. (C) Muscle fiber (Patient 7, *POLG*) with segmental COX deficiency spread through approximately 70% of the cell's longest diameter. (D) Muscle fiber (Patient 7, *POLG*) with complete COX deficiency. All examples in A–D are from Patient 7 (POLG). (E) Quantification of mitochondrial content based on SDHA fluorescence intensity in COX‐deficient foci and in matched COX‐positive subsarcolemmal regions within individual muscle fibers. Data are combined from Patients 3 (*RRM2B*), 5 (*POLG*), and 7 (*POLG*). Data are plotted for each cell relative to COX‐positive areas (n = 74, 2‐tailed paired *t* test, *p* < 0.0001). (F) Correlation between SDHA and VDAC1 (porin) are plotted against each other for a sample of muscle fibers, the regression line has an *r* ^2^ = 0.644 and *p* < 0.0001. Scale bars = 25 µm.

When surveying COX/SDH histochemistry, we consistently found that focal regions of deficiency are localized in the subsarcolemmal space (see Fig 1B–D). This finding was corroborated by quantitative immunofluorescence (Fig 2).^19^ We generated MTCOI/SDHA intensity profiles to map the distribution of COX deficiency with subcellular resolution. The spectrum of MTCOI deficiency ranged from confined perinuclear foci to segmental and completely MTCOI‐deficient muscle fibers (Fig 2A–D). Furthermore, comparing SDHA fluorescent intensity in COX‐deficient foci and COX‐positive regions of the same fibers indicated that SDHA protein abundance was consistently higher by an average of 3.3‐fold in MTCOI‐deficient foci (see Fig 2E), indicating a local increase of mitochondrial mass specifically in areas of respiratory chain deficiency.

We verified that this increase in SDHA protein was specific to COX‐deficient niches and not a result of increased mitochondrial content near the nucleus (ie, perinuclear vs subsarcolemmal) by analysis of normal muscle fibers from patients and controls (data not shown). The use of SDHA as a mitochondrial mass marker was also validated against VDAC1 (see Fig 2F), showing that both markers yield equivalent results.

### Respiratory‐Deficient Foci Contain mtDNA Deletions

**Figure 3:**
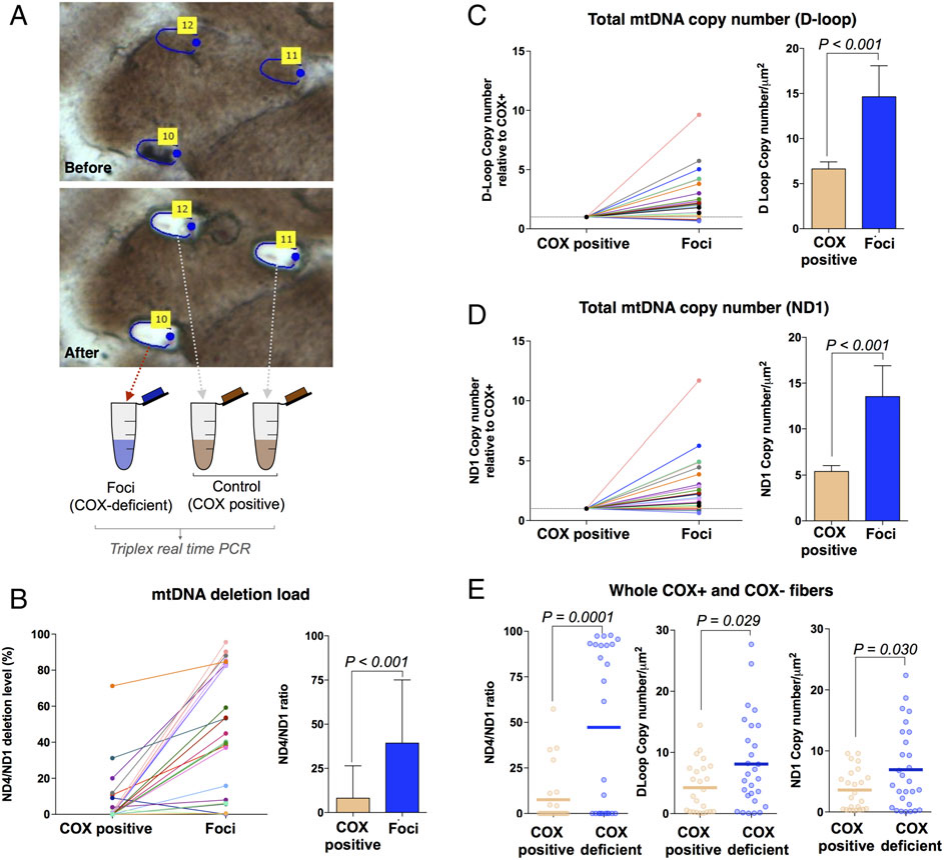
Cytochrome c oxidase (COX)‐deficient foci contain high levels of mtDNA deletions and show compensatory increase in mtDNA copy number. Subcellular and single cell mtDNA analysis was performed on patients with mutations in *POLG* (n = 2), *RRM2B* (n = 2), and *TWNK* (n = 1; Patients 2–5 and 7). (A) Single muscle fiber before and after laser microdissection of 1 COX‐deficient and 2 COX‐positive (COX+) control subcellular regions. (B) ND4/ND1 ratio as an indicator of mtDNA deletion level in COX‐deficient foci with matched COX‐positive subcellular regions from single fibers (left) and mean deletion level ± standard error of the mean (SEM; right). (C) Total D‐Loop mtDNA copy number in COX‐deficient foci and matched COX‐positive subcellular regions from single fibers. mtDNA copy numbers are shown relative to COX‐positive regions of the same muscle fiber (left) and average copy number by group (right). Mean copy number ± SEM is shown (right). (D) The same as C but with ND1 as the copy number metric. (E) mtDNA ND4/ND1 ratio (left), total D‐Loop (center), and total ND1 (right) mtDNA copy number in COX‐deficient and COX‐positive whole fibers isolated by laser capture microdissection. A value of 0 indicates no ND4 deletion, whereas a value of 100 indicates all mtDNA molecules contain a ND4 deletion (left). Each datapoint corresponds to a single fiber. Bars indicate mean values. Matched subcellular regions in B–D are connected by a matched‐colored line. n = 27 fibers, 5 patients, 2‐tailed paired *t* tests.

We next confirmed that respiratory chain–deficient foci contain higher levels of mtDNA deletions than the rest of the muscle fiber, using laser capture microdissection of small subcellular areas followed by triplex real‐time PCR (Fig 3). This analysis was performed in patients with mutations in mtDNA maintenance genes *POLG or RRM2B* (Patients 2–5 and 7). COX‐deficient foci and 2 identically sized COX‐positive areas of the same muscle fiber were microdissected (see Fig 3A).

The majority of COX‐deficient foci had deletions encompassing *MT‐ND4*, and some deletions encompassing *MT‐ND1*, whereas COX‐positive areas of the same muscle fiber generally only contained normal mtDNA, or lower levels of the deletion (see Fig 3B). On average, significantly higher mtDNA deletion levels were detected in foci compared to COX‐positive regions. Less than 10% of fibers had deletions encompassing *MT‐ND1* (not shown), consistent with previous findings.^27^ The same deletion pattern was observed in whole fibers, consistent with foci and fully deficient fibers being etiologically related.

### mtDNA Copy Number and Mitochondrial Mass Is Increased in COX‐Deficient Foci

If increased mitochondrial biogenesis contributed to the accumulation of deleted mtDNA molecules, we reasoned that mtDNA copy number should similarly be elevated in the respiratory chain–deficient foci compared to COX‐positive regions of the same cell. Therefore, we measured absolute mtDNA copy number density in COX‐deficient foci relative to corresponding COX‐positive regions of the same fibers, using an optimized standard curve method.^17^ Because *MT*‐*ND4* and occasionally *MT‐ND1* can both be deleted in human skeletal muscle,^27^ we used D‐Loop and *MT‐ND1* amplicons as metrics of copy number.

Consistent with the elevation in mitochondrial protein content (see Fig 2E), mtDNA copy number was increased in the majority of COX‐deficient foci (see Fig 3C–D). Isolated COX‐deficient foci relative to COX‐positive regions showed a mean 2.2‐fold higher (range = 1.1–9.6, *p* < 0.001) D‐Loop copy number. This was confirmed by *MT‐ND1* copy number, where the within‐fiber difference between COX‐deficient and COX‐positive regions was 2.5‐fold (range = 1.1–11.7, *p* < 0.001). In comparison, cells with complete COX deficiency showed on average a 1.9‐fold (range = 1.0–6.5, *p* = 0.03) higher D‐Loop copy number and 1.9‐fold (range = 1.0–6.2, *p* = 0.03) higher *MT‐ND1* copy number compared to cells with normal COX activity (see Fig 3E). Thus, COX‐deficient foci are associated with local upregulation of mtDNA copy number and mass.

### Accumulation of COX‐Deficient Mitochondrial Mass Occurs Near Myonuclei

**Figure 4:**
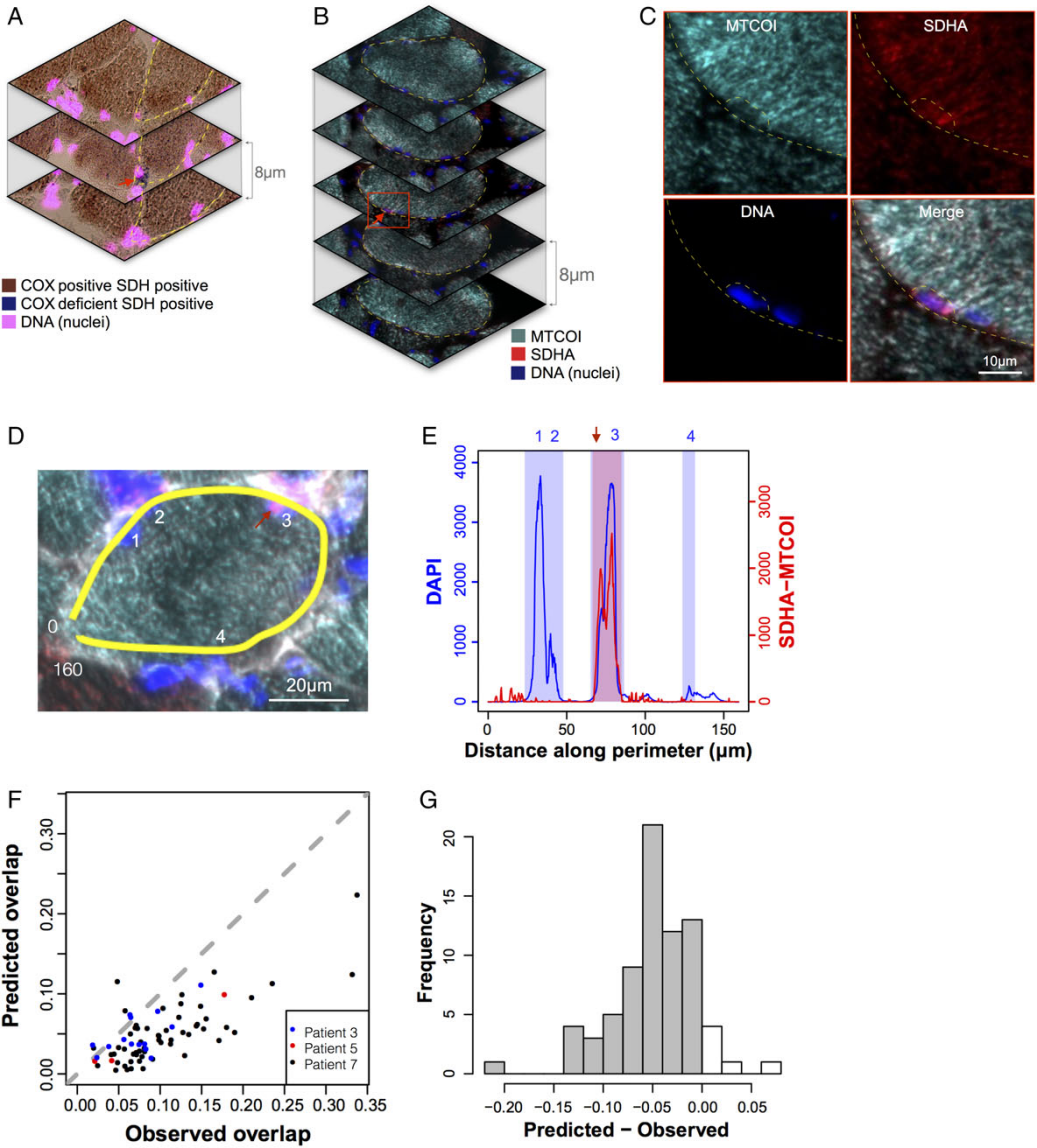
Foci of cytochrome c oxidase (COX)‐deficient mitochondria are located in the subsarcolemmal region and colocalize with myonuclei. (A–C) Serial cryosections from Patient 7 with recessive *POLG* mutations (POLG), (A) reacted for sequential COX/succinate dehydrogenase (SDH) histochemistry and 4,6‐diamidino‐2‐phenylindole (DAPI) and (B) labeled by triple immunofluorescence for mitochondrial cytochrome c oxidase submit 1(MTCOI), SDH complex subunit A (SDHA), and DAPI. Note the focal area of COX deficiency in the outlined cell restricted to the middle section *(red arrow)*, indicating that the focus is <8 µm in length. (C) Magnified area from B showing selective absence of MTCOI staining in the perinuclear niche area outlined. (D) An example muscle fiber from Patient 3 with recessive *RRM2B* mutations (RRM2B), analyzed using a perimeter line scan (yellow). Nuclei are indicated with numbers and the focal area of COX deficiency with a red arrow. The line scan goes from 0 to 160 µm. (E) Arbitrary fluorescence intensity for DAPI and SDHA minus MTCOI along the perimeter line scan from D. Shaded areas represent perinuclear areas (blue) and COX‐deficient focal area (red) used to compute the degree of overlap. Note that the COX‐deficient area overlaps with nucleus 3. The same analysis was applied to all foci. (F) Scatterplot comparing predicted and observed overlap of COX‐deficient areas and nuclei along the perimeters of muscle fibers with COX‐deficient foci. Data are from Patients 3 (*RRM2B)*, 5 (*POLG*), and 7 (*POLG*), expressed as fractions of total muscle fiber perimeter length. Note the distribution of data toward the right of the dotted line, which represents chance level (observed = expected). (G) Frequency histogram of predicted − observed overlap fractions; n = 74, 1‐sample *t* test, *p* = 4.26e^−15^.

Mitochondrial biogenesis is dependent on several nuclear encoded proteins; therefore, we hypothesized that the localization of COX‐deficient mutant niches in the subsarcolemmal space may be due to the presence of myonuclei. Thus, serial sections were subjected to COX/SDH histochemistry counterstained with DAPI to determine the focal distribution and topology of COX‐deficient foci in relation to the myonuclei along the length of muscle fibers (Fig 4A). Immunofluorescent labeling of serial sections for MTCOI/SDHA/DAPI objectively confirmed the colocalization of COX‐deficient niches with myonuclei (see Fig 4B, C).

To identify COX‐deficient foci that did not colocalize with nuclei, we profiled perinuclear regions and MTCOI fluorescent intensity along the perimeters of fiber sections containing a region of COX deficiency. Perinuclear regions were identified automatically by thresholding DAPI signal along the perimeter. Focal regions of COX deficiency were manually classified by visual inspection of SDHA and MTCOI profiles along the perimeter (see Fig 4D, E) and by line scan quantification as in Figure 2. Across 74 fiber sections containing COX‐deficient foci, 26.2% of the perimeter was classified as perinuclear and 20.0% was classified as COX‐deficient foci. Given the relative proportions of perinuclear space and focal deficiency, if the location of nuclei and COX‐deficient foci were unrelated (ie, random), we would expect only 5.23% of the perimeter to be classified as overlap. In contrast, 9.5% was classified as COX‐deficient foci and perinuclear overlap. To determine the significance of this difference, in each muscle fiber the observed overlap was measured and the predicted foci–nuclei overlap was calculated.

The differences between predicted and observed overlap fractions were found to be significantly lower than zero (*p* = 4.26e^−15^; see Fig 4F, G). A total of 75 distinct foci were identified in 74 cells, all of which at least partially overlapped with a perinuclear region, consistent with the required presence of a nucleus for clonal expansion of mutant mitochondria.

### Localized Activation of Mitonuclear Signaling Pathways with mtDNA Deletions

**Figure 5:**
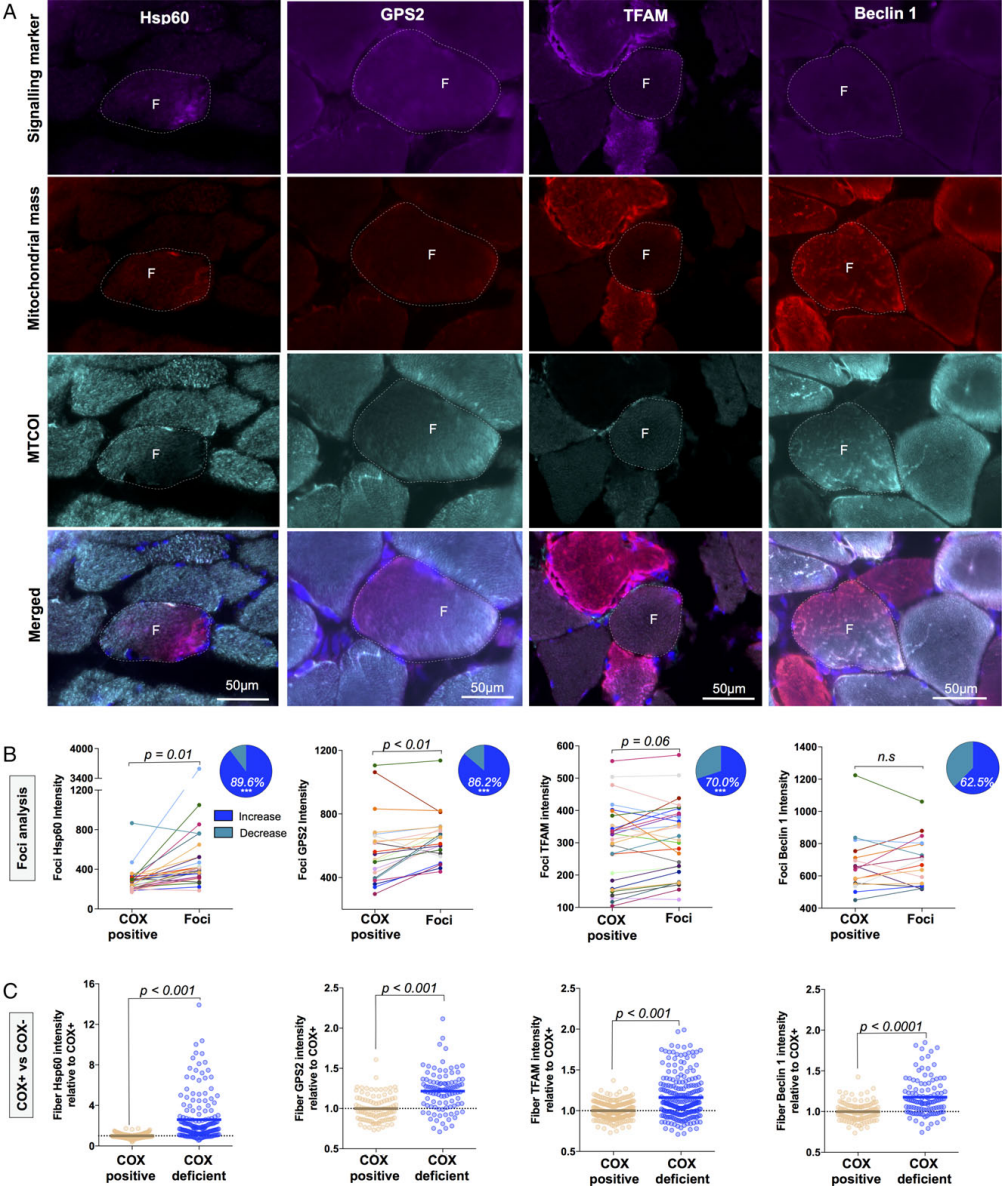
Activation of mitonuclear signaling pathways in response to subcellular cytochrome c oxidase (COX) deficiency. (A) Quadruple immunofluorescent imaging of mitonuclear signaling targets Hsp60 (Patient 7, *POLG)*, GPS2 (Patient 5, *POLG*), transcription factor A, mitochondrial (TFAM; Patient 7, *POLG*), and Beclin1 (Patient 5, *POLG*) in combination with anti–mitochondrial cytochrome c oxidase subunit I (MTCOI), anti–succinate dehydrogenase complex subunit A (SDHA), or anti‐VDAC1 (for Hsp60) and 4,6‐diamidino‐2‐phenylindole. F indicates COX‐deficient foci, and dashed lines indicate the fiber boundaries. (B) Subcellular quantification of fluorescent intensity in COX‐deficient foci compared to COX‐positive areas of the same cell from Patient 3 (*RRM2B*), Patient 5 (*POLG*), and Patient 7 (*POLG*). Values from the same cell are connected by a line; n = 29 (Hsp60), n = 29 (GPS2), n = 30 (TFAM), and n = 16 (Beclin1); paired *t* test. Inset: Pie charts represent the percentage of foci that have an increase in signal relative to COX‐positive areas. ***p < 0.0001, Wilson/Brown binomial test comparing each percentage to the null hypothesis of 50:50. (C) Whole cell fluorescent intensity in full COX‐positive and COX‐deficient fibers from Patient 3 (*RRM2B*), Patient 5 (*POLG*), and Patient 7 (*POLG*). Each datapoint represents a muscle fiber; n = 175 (Hsp60), n = 101 (GPS2), n = 230 (TFAM), n = 112 (Beclin1); Mann–Whitney test. Data are from Patients 3 (RRM2B), 5 (POLG), and 7 (POLG).

To assess whether signaling may be contributing to the increased mitochondrial content and mtDNA deletions in the perinuclear niche, we probed markers of (1) mitochondrial biogenesis, (2) mitochondrial unfolded protein response (UPR^mt^), and (3) mitophagy (Fig 5). We analyzed the mitochondrial biogenesis marker TFAM, UPR^mt^ marker Hsp60, retrograde signaling factor GPS2,^28^ and mitophagy marker Beclin1. COX‐deficient foci, representing early stage respiratory chain deficiency, were compared to equivalent size COX‐positive regions of the same fibers. Fully COX‐deficient fibers, representing the late stage respiratory chain deficiency, were compared to COX‐positive fibers.

Relative to COX‐positive regions of individual muscle fibers, Hsp60 and GPS2 were both higher in focal regions of deficiency for 89.6% and 86.2% of foci, respectively (see Fig 5B). Both the UPR^mt^ and GPS2 retrograde signaling pathways have been shown to be upstream of mitochondrial biogenesis.^12^, ^28^ Accordingly, mitochondrial TFAM was also increased for 70% of foci relative to COX‐positive regions. In fully COX‐deficient fibers, Hsp60 and GPS2 were also significantly increased relative to COX‐positive fibers (see Fig 5C). TFAM was significantly elevated in fully COX‐deficient fibers, consistent with the overall increase in mtDNA copy number in late stage COX‐deficient fibers.

To verify that these results were not driven by the proximity of foci to nuclei in the SS area, we also analyzed COX‐positive perinuclear regions from COX‐positive fibers of patients and controls, which were compared to subsarcolemmal but nonperinuclear regions. We found that Hsp60 and TFAM levels were similar; however, GPS2 was elevated in perinuclear regions, consistent with its role as nuclear transcription factor.^28^ In patient muscle, compared to perinuclear COX‐positive areas, Hsp60 (2.2‐fold), GPS2 (1.2‐fold), and TFAM (1.5‐fold) levels were all higher in COX‐deficient niches, confirming the specificity of this finding.

A reduction in quality control processes to remove mutant mitochondria via autophagy could also have contributed to the accumulation of mutant mitochondria in specific subcellular compartments. Immunolabeling of the autophagic regulator Beclin1^29^ showed that levels were unchanged in COX‐deficient foci, although significantly higher in fully COX‐deficient fibers (see Fig 5B, C), with no difference between the SS and IMF region.

### Respiratory Chain Deficiency First Spreads in Cross‐Section following Mitochondrial Network Anisotropy

**Figure 6:**
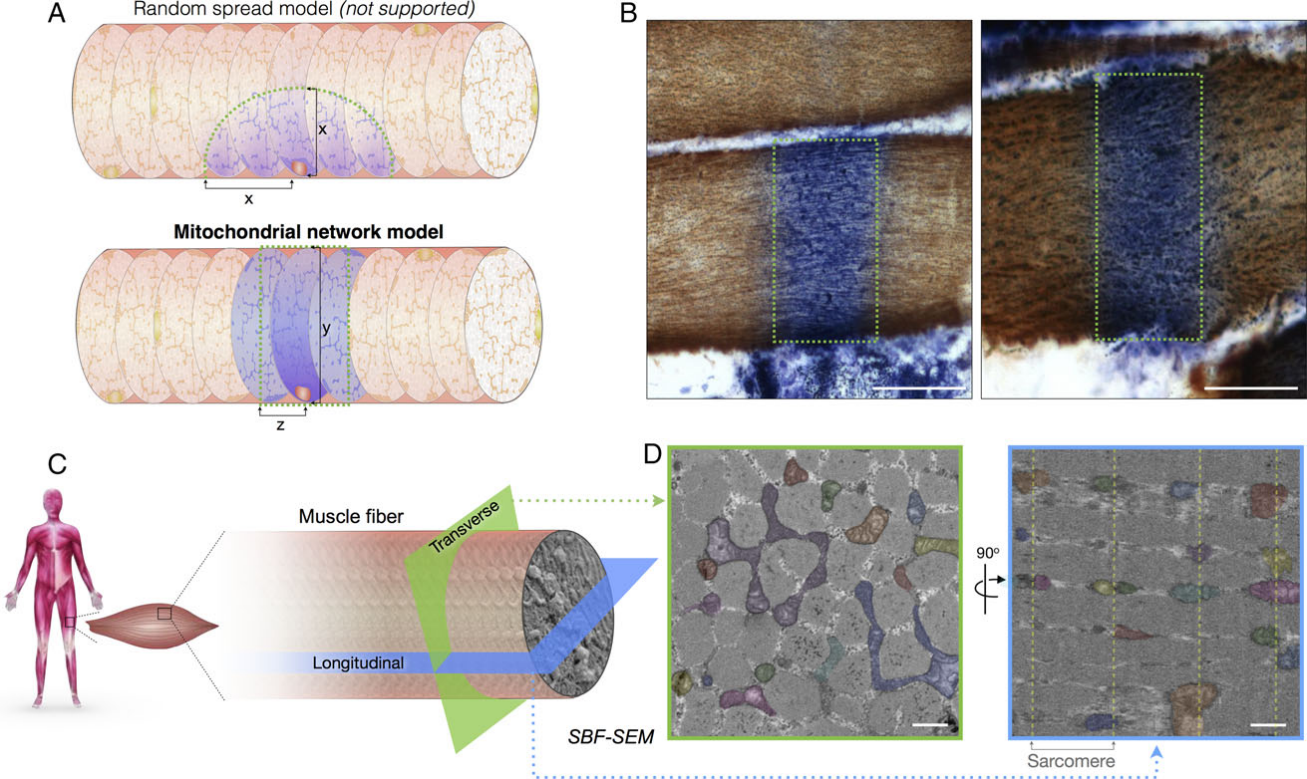
Subcellular patterns of cytochrome c oxidase (COX) deficiency and preferential transverse mitochondrial network connectivity in human muscle. (A) Hypothetical models illustrating potential spread of COX‐deficient succinate dehydrogenase (SDH)‐positive foci. In the random spread model (top), biochemical deficiency covers an equal distance (x) in both transverse and longitudinal orientations. In the mitochondrial network model (bottom), COX negativity can cover the width of the muscle fiber, a distance (y) that is greater than the distance covered in the longitudinal axis of the fiber (z). Dotted green areas denote edges of biochemical deficiency. (B) Images of thin longitudinal regions of COX‐deficient segments in COX/SDH histochemistry, supporting the mitochondrial network model. (C) Schematic demonstrating differences in mitochondrial network connectivity when a muscle fiber is examined in transverse or longitudinal orientation. (D) Results from serial block face scanning electron microscopy showing that in the transverse orientation (green), mitochondria form an interconnected network. In the longitudinal orientation (blue), mitochondria appear round and isolated, with few connections across sarcomeres. Each continuous mitochondrion is pseudocolored and Z‐lines are marked by dotted lines. Scale bars for histochemistry (B) = 50 μm; scale bars for electron microscopy (D) = 1 μm.

Finally, we sought to address whether mitochondrial network connectivity may dictate the segmental appearance of respiratory chain deficiency (Fig 6). Examining muscle fibers longitudinally revealed the existence of strikingly short COX‐deficient muscle fiber segments, where the diameter of the COX‐deficient muscle fibers is substantially wider than the longitudinal axis.

In skeletal muscle cells, intermyofibrillar mitochondria organized at the Z‐bands show a substantial degree of branching. We examined the 3‐dimensional morphology of skeletal muscle mitochondria in human controls (n = 8) and patients (n = 6) using SBF‐SEM (see Fig 6C, D). Controls and patients both demonstrate high mitochondrial connectivity across the transverse orientation of each muscle fiber and low connectivity between Z‐bands along the muscle fiber; representative images are shown in Figure 6D. These results support the mitochondrial network model and likely provide the structural basis for the segmental COX deficiency regularly observed in mitochondrial myopathy and aging.^30^

### Foci Are Observed in Aging and Other Neuromuscular Conditions

**Figure 7:**
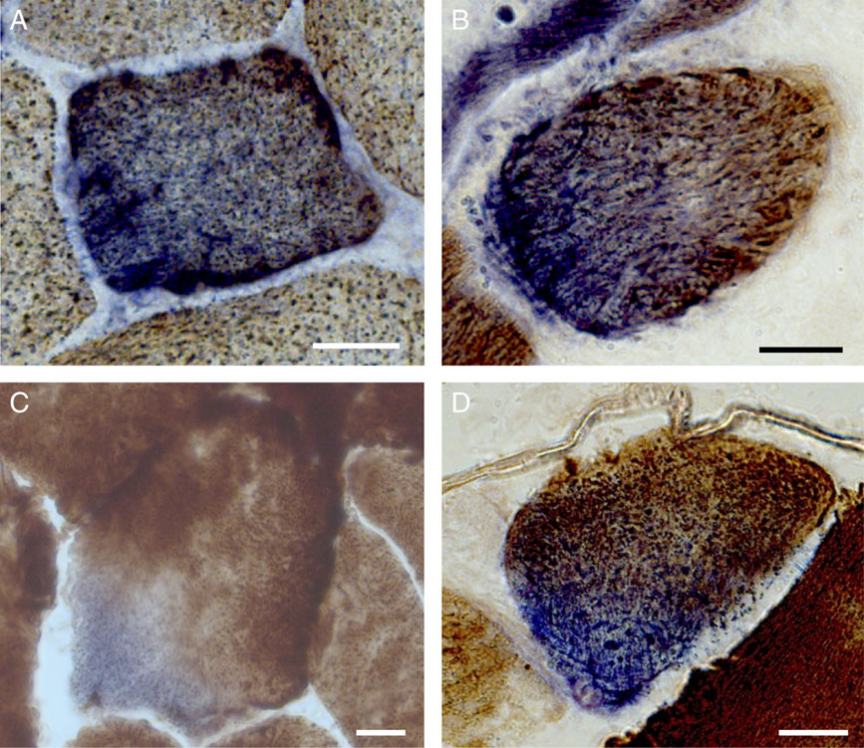
Sub‐cellular foci identified in other neuromuscular diseases and aging from cytochrome c oxidase (COX)/succinate dehydrogenase (SDH) histochemistry. Focal COX‐deficient SDH‐positive region in cross‐section from cases of (A) single, large‐scale mtDNA deletion, (B) inclusion body myositis, (C) mechanically ventilated diaphragm, and (D) normal aging skeletal muscle. Scale bars = 25 μm.

In addition to mtDNA maintenance disorders, we sought to determine whether foci of deficiency are observed in other myopathies in which de novo mtDNA deletions and COX deficiency are also found. COX‐deficient foci were also identified in cases of single, large‐scale mtDNA deletions, inclusion body myositis, and mechanically ventilated diaphragm, where COX deficiency is associated with mtDNA deletions (Fig 7).^26^ Furthermore, muscle from healthy adults 68 to 77 years of age (n = 79) also contained COX‐deficient foci, which suggested that the observation is not specific to mitochondrial myopathy but is likely relevant to other age‐related myopathic conditions.

## Discussion

Mitochondrial respiratory chain deficiency in mtDNA maintenance disorders is dependent on the progressive accumulation of mtDNA deletions. Several theories have been proposed to explain the clonal expansion of mtDNA deletions.^4^, ^5^, ^6^, ^7^ However, a number of questions remain. In postmitotic muscle fibers, where do mtDNA deletions originate? How do deleted mtDNA molecules accumulate to higher levels than wild‐type mtDNA? What dictates the directional spread of respiratory chain–deficient segments along muscle fibers?

To explore these questions, we performed in‐depth imaging and molecular studies of skeletal muscle biopsies from patients with genetically confirmed mtDNA maintenance disorders. We targeted the smallest portion of the muscle fiber presenting with COX deficiency and found that these areas are always perinuclear. In addition, we have shown that there is accumulation of mtDNA deletions in the COX‐deficient foci along with corresponding increase in nuclear‐mitochondrial signaling. Therefore, we propose that mtDNA deletions arise and accumulate within the perinuclear mitochondria, where nuclear proximity of mtDNA deletions and local induction of biogenesis are key factors for clonal expansion.

### Nuclear Proximity as a Determining Factor in Focal COX Deficiency

Three factors could contribute to explain why mtDNA deletions and respiratory chain deficiency originate in perinuclear mitochondria: (1) increased mutation rate in the perinuclear area, (2) higher replication rates in the perinuclear area, or (3) proximity to the nucleus for retrograde mitonuclear signaling. Davis and Clayton^31^ have previously demonstrated that mtDNA replication rate is higher in the perinuclear region compared to the rest of the cell, consistent with the requirement of nuclear gene products for mitochondrial biogenesis.

In patients harboring mutations in nuclear DNA genes encoding mtDNA replication machinery, a higher local replication rate could account for a higher mutation rate among perinuclear mitochondria. Thus, it is also possible that there may be multiple deletion species competing within a single focus and that the chance of mtDNA deletions being replicated in the perinuclear region may also be higher, due to the higher replication rate.

Furthermore, our data demonstrate that focal regions of COX deficiency have elevated mitochondrial mass and mtDNA copy number concomitant with higher levels of mitonuclear signaling factors, indicative of mitochondrial biogenesis through mitonuclear “retrograde” signaling. In *Caenorhabditis elegans*, mtDNA deletions trigger nuclear activation of the unfolded protein response (UPR^mt^) to promote mitochondrial biogenesis and mitochondrial dynamics.^11^, ^12^ With mtDNA deletions existing throughout the worm from birth, this process may increase mtDNA heteroplasmy.^11^, ^12^ The UPR^mt^ has also been shown to modulate both biogenesis and mitophagy levels.^11^, ^12^ Therefore, given the observations in *C. elegans*, it is possible that the UPR^mt^ in foci of human muscle may be linked with activation of mitochondrial biogenesis.

Our data in human skeletal muscle suggest a role for the UPR^mt^ and possibly implicate other factors such as GPS2, which transcriptionally activates mitochondrial biogenesis in response to mitochondrial stress or depolarization.^28^ Beclin1 labeling suggests no change in mitophagy in small foci representing the early stage of deficiency. However, although Beclin 1 was the only target for which antibodies of sufficient quality on frozen muscle samples was available, Beclin1 may not reflect all mitophagy pathways. Based on the differential upregulation of signaling factors in focal COX‐deficient and fully COX‐deficient fibers, it remains possible that multiple signaling pathways are activated at different stages of expansion and in different fibers types. Clinically meaningful models will be required to generate mechanistic data and fully resolve these questions.

### Focal Deficiency, Clonal Expansion, and Mitochondrial Disease Progression

In addition to representing a novel pathological feature of mtDNA maintenance disorders, focal regions of deficiency were also observed in muscle from patients with single, large‐scale mtDNA deletions and in normal aging. Segmental respiratory chain deficiency in longitudinal muscle fibers is a pathological hallmark of mitochondrial myopathy and aging.^3^, ^24^, ^30^ To our knowledge, this is the first study to report circumscribed cytoplasmic areas of COX deficiency smaller than the diameter of muscle fibers. Our data also demonstrate that, in a muscle section, foci are relatively infrequent compared to fully COX‐deficient muscle fibers. Furthermore, although COX‐deficient segments in longitudinal orientation have been known to vary greatly in length, this is the first report demonstrating segments that are substantially shorter than the width of the muscle fiber (see Fig 6B).

Applying SBF‐SEM analysis for the first time in human muscle confirmed the substantially more elaborate nature of the mitochondrial network along Z‐bands (ie, I‐band mitochondria) in the transverse orientation of human muscle fibers, compared to sparse longitudinal interconnections between sarcomeric planes (see Fig 6D). This is in contrast to the almost continuous mitochondrial network previously reported in mice.^32^ Because contiguous mitochondria can exchange (mutant) mtDNA through fusion, the anisotropic nature of the human mitochondrial network may account for COX‐deficient segments that appear restricted along the length of affected muscle fibers.

### Conclusions and Proposed Model

Based on our observations, we propose the following model. mtDNA deletions that arise in the perinuclear region preferentially accumulate under the influence of mitochondrial biogenesis, reaching critical mass and causing the first visible signs of focal mitochondrial respiratory chain deficiency. Mutant mitochondria then preferentially propagate transversely via direct physical interactions between mitochondria, driven in part by increased local mitochondrial biogenesis triggered by retrograde signaling from mutant mitochondria to the surrounding nucleus. The clonal nature of these deletions and the role that mtDNA replication plays in this process remain to be investigated. This hypothesis assumes that fully COX‐deficient cells (which must have a point of origin) are derived from the spread of small proliferative perinuclear foci. Human‐relevant disease models where this pathogenic process can be observed over time will be needed to confirm this mechanism, and to determine if it can be targeted therapeutically to prevent the clonal accumulation of mtDNA defects.

## Author Contributions

A.E.V., M.P., and D.M.T. contributed to the conception and design of the study. A.E.V., H.S.R., K.P., C.L., C.Ch., A.G., K.W., T.D., D.D., G.F., K.A.R., M.C.R., A.K.R., R.W.T., V.P., B.J.P., A.A.S., and C.Co. contributed to acquisition of data and data analysis. A.E.V., H.S.R., C.L., M.P., and D.M.T. contributed to writing the manuscript.

## Potential Conflicts of Interest

Nothing to report.

## Supporting information

Supporting Information
