# Supplementary material for: Subcellular origin of mitochondrial DNA deletions in human skeletal muscle
Source: Ann Neurol. 2018 Aug 21;84(2):289–301. doi: 10.1002/ana.25288 (PMC6141001; doi:10.1002/ana.25288)
Supplement: Supplementary file 1 — Supporting Information [file ANA-84-289-s001.docx]

**Supplementary Table 1** Case information for patients examined including patients with multiple mtDNA deletions (n=7). COX negative fibres are those deficient for COX activity but positive for SDH activity (COX- SDH+). Ragged red fibers (RRFs) are fibers deficient for COX activity with excess SDH activity (COX- SDH++).

| **Patient #** | **Gender** | **Age at onset** | **Age at biopsy** | **Gene** | **Mutation** | **% COX deficiency** | **% RRF** | **Clinical phenotype** | **Reported previously?** |
| --- | --- | --- | --- | --- | --- | --- | --- | --- | --- |
| 1 | F | N/A | 31 | *TWNK* | p.(Arg374Gln) | 10% | 1% | adPEO | nr |
| 2 | M | 14 | 37 | *RRM2B* | p.(Thr141Ile); p.(Gly273Ser) | >40% | 20% | arPEO, ptosis, proximal muscle weakness | Pitceathly et al. Brain (2012), Patient 19 |
| 3 | F | 11 | 43 | *RRM2B* | p.(Arg186Gly); p.(Thr218Ile) | 30% | 5% | arPEO, asymmetrical ptosis, distal and proximal muscle weakness plus | Pitceathly et al. Brain (2012), Patient 20 |
| 4 | F | 38 | 64 | *RRM2B* | p.(Asp70Asn) | 8% | 3% | adPEO, ptosis, ataxia, proximal muscle weakness | Pitceathly et al. Brain (2012), Patient 5 |
| 5 | F | N/A | 66 | *POLG* | *POLG*:p.(Ala467Thr); p.(Ser933Arg) | 24% | 6% | arPEO, ataxia | nr |
| 6 | F | N/A | 69 | *TWNK* | p.(Arg303Trp) | 30% | 8% | adPEO, ptosis | nr |
| 7 | M | N/A | 80 | *POLG* | p.(Thr251Ile)/p.(Pro587 Leu); p.(Ala467Thr) | 20% | 4% | arPEO | nr |

adPEO, autosomal dominant progressive external ophthalmoplegia; arPEO, autosomal recessive progressive external ophthamoplegia;

CPEO, chronic progressive external ophthalmoplegia; nr, not reported
